# Very high vitamin D supplementation rates among infants aged 2 months in Vancouver and Richmond, British Columbia, Canada

**DOI:** 10.1186/1471-2458-11-905

**Published:** 2011-12-07

**Authors:** Barbara Crocker, Tim J Green, Susan I Barr, Bridgid Beckingham, Radhika Bhagat, Beata Dabrowska, Rachel Douthwaite, Carmen Evanson, Russell Friesen, Kathy Hydamaka, Wangyang Li, Kelly Simmons, Lillian Tse

**Affiliations:** 1 Infant, Child and Youth Program, Vancouver Coastal Health, 1669 East Broadway, Vancouver V5N 1V9, Canada; 2 Food, Nutrition and Health, University of British Columbia, 2205 East Mall, Vancouver V6T 1Z4, Canada; 3 Healthy Babies and Families Program, Vancouver Coastal Health, 8100 Granville Avenue, Richmond V6Y 3T6, Canada

## Abstract

**Background:**

Vitamin D deficiency during infancy may lead to rickets and possibly other poor health outcomes. The World Health Organization recommends exclusive breastfeeding for the first 6 months. Breast milk is the best food for infants but does not contain adequate vitamin D. Health Canada recommends all breastfed infants receive a daily vitamin D supplement of 400 IU; however, there appears to be limited current Canadian data as to whether parents or caregivers are following this advice. The aim of this study was to determine the rates of vitamin D supplementation among 2-month old infants in Vancouver and Richmond, British Columbia, Canada.

**Methods:**

Mothers of all healthy infants born between April and May 2010 were approached to participate. Telephone surveys were conducted with 577 mothers (response rate 56%) when their infants turned 2 months.

**Results:**

Over half of the infants received only breast milk in the week prior to the survey. One third received a mixture of breast milk and infant formula and 10% received only formula. About 80% of the infants were supplemented with vitamin D at 2 months. Infants who received only breast milk were most likely to be supplemented with vitamin D (91%). Over 60% of the infants had a total vitamin D intake of 300- < 500 IU/d from supplements and formula and only 5% did not receive any vitamin D. Most parents were advised to give vitamin D supplement by health professionals, such as public health nurses, midwives, and doctors.

**Conclusions:**

About 90% of the infants received breast milk at 2 months of age. The vitamin D supplementation rate was 80%. Future studies are needed to monitor breastfeeding duration and vitamin D supplementation rates as infants get older.

## Background

Worldwide public health authorities recommend exclusive breastfeeding for the first 6 months of life for healthy term infants [1-3]. Breast milk is the best food for optimal growth of the infant and breastfeeding has been associated with improved health outcomes for mother and infant [1]. While breast milk is the ideal food for infant, it does not generally supply adequate amounts of vitamin D [4]. As such, breastfed infants are at risk of vitamin D deficiency [5]. In its most serious form vitamin D deficiency in infancy leads to rickets, a condition characterized by weakened bones, resulting from poor mineralization of newly formed bone tissue [6]. Additionally, there is emerging evidence that lack of vitamin D during infancy, is associated with altered calcium metabolism [7], early childhood tooth decay [8], and increased risk of Type 1 Diabetes [9], and asthma later on [10].

While infant formula is fortified with vitamin D, breastfed infants are reliant on skin synthesis of vitamin D through the action of sunlight or supplemental vitamin D. Due to the risk of skin cancer it is generally recommended that infants under 1 year be kept out of direct sunlight [11]. Owing to concerns about vitamin D deficiency, Health Canada [4], the American Academy of Pediatrics [3], as well as several European countries [12-14] recommend that breastfed infants receive a daily vitamin D supplement, usually 400 IU. In Canada this recommendation has been in place since 1967 [4], yet, other than in the province of Quebec [15,16], there are few data as to whether parents or caregivers are following this advice. As part of the Canadian Community Health Survey 2007-2008 [17], Statistics Canada reported that among women who had given birth in the past 5 years and exclusively breastfed their infant, 67% provided a vitamin D supplement to the infant. However, this survey did not consider the frequency, dose, or form of supplement, nor were supplementation practices assessed among women who fed their infants a combination of breast milk and formula. Further, this survey included women who gave birth as early as 2002 and there may now be greater awareness about the importance of vitamin D. Indeed, Gallo et al. [18] reported that in one Montreal hospital 98% of exclusively breastfeeding mothers who gave birth in 2007-2008 supplemented their infants at some point prior to 6 months of age.

As part of British Columbia's (BC) publicly funded medical system, public health nurses provide breastfeeding support including advice on vitamin D supplementation within the first few days postpartum. To assess the effectiveness of this health promotion strategy, we conducted a survey in 2010 to determine the rates of breastfeeding and vitamin D supplementation among 2-month old infants in Vancouver and Richmond, BC. We also wanted to determine the type and dose of vitamin D supplementation given, examine the association of socio-demographic factors and vitamin D supplementation and determine barriers to infant vitamin D supplementation.

## Methods

### Sampling frame

Ethical approval to conduct the study was obtained from the University of British Columbia Behavioural Research Ethics Board. The survey was conducted in Richmond and five of six Community Health Areas in Vancouver. Vancouver (population 578,041; 2006) and Richmond (population 174,461; 2006) are the largest and fourth largest cities, respectively, in BC, Canada. All parents or caregivers whose infant was born between April and May of 2010 were invited to participate in the survey. Parents or caregivers were ineligible: if they moved out of the catchment area or were involved in a program that provided care to high-risk mothers (i.e. substance-abuse); or if their infant was adopted, in foster-care, or under the care of a neonatal intensive care unit. An infant age of 2 months was chosen because we wanted to survey women who had established breastfeeding and before the breastfeeding rates decline. Initial recruitment took place through public health nurses who asked the parent at an early postpartum contact whether they would be willing to participate in a short telephone interview. At an infant age of 2 months (range = 7-10 weeks) a research assistant phoned the parent to complete the survey after again obtaining verbal consent to participate.

### Survey

The survey questionnaire was designed to obtain information on infant feeding practices; vitamin D supplementation including the form, frequency, and dose; who recommended that the infant be supplemented and (if appropriate) the reasons for not supplementing; and basic socio-demographic questions. The questionnaire was initially developed by the study investigators through face-to face meetings and through consultation and focus testing with relevant stakeholders. We piloted the questionnaire with ten new parents and revised it accordingly. The questionnaire was administered in English or when required in Mandarin, Cantonese, Punjabi, Vietnamese, or Spanish.

### Data analysis

In our study, we defined "All breast milk" as infants who had received only breast milk (vitamins, minerals, and medicines permitted) in the week prior to the survey [2]. "Mixed breast milk and formula" was used to define infants who had received a mixture of breast milk and infant formula in the week prior to the survey. "Infant formula" was used to define infants who had received only formula in the week prior to the survey. Based on data indicating that 2-month old infants are fed an average of 8 times per day [3], we further divided the "mixed breast milk and formula" group into: "≥75% of breast milk" defined as ≤2 feedings of other liquids/food per day; "50- < 75% breast milk" as 3-4 feedings of other liquids/food per day; and " < 50% breast milk" as > 4 feedings of other liquids/food per day. Average daily formula intake was calculated by multiplying frequency of consumption in 24 hours by the amount of formula received in each bottle feed. The amount of vitamin D provided by formula was calculated assuming infant formula contains 40 IU vitamin D/100 ml, and the average intake of vitamin D provided by supplements was calculated based on the amount per dose and the frequency of administration. These two sources were summed to determine daily vitamin D intake. Descriptive statistics were used to describe breastfeeding and vitamin D supplementation rates (proportion with 95% confidence interval). Chi Square tests were used to determine differences in vitamin D supplementation by feeding practice. Rates were also stratified by socio-demographic variables such as ethnicity. The proportion of infants receiving a total vitamin D intake of < 300 IU from supplements and/or formula was also examined by feeding practice using Chi-square. A cut-point of 300 IU was selected because in order to achieve 400 IU per day a caregiver of breastfed infant would need to be 100% compliant with daily supplementation during the week surveyed. Further, the evidence base for infant requirements is poor [19] and recent trials suggest that 400 IU vitamin D exceeds requirements [20]. Multiple logistic regression was used to determine significant predictors (from among age, ethnicity, parity, income, and education) of receiving vitamin D supplements in two separate models; one in women whose infants had only received breast milk in the week prior to the survey and the other in those providing at least 50% of feeds from breast milk.

## Results

**Figure 1:**
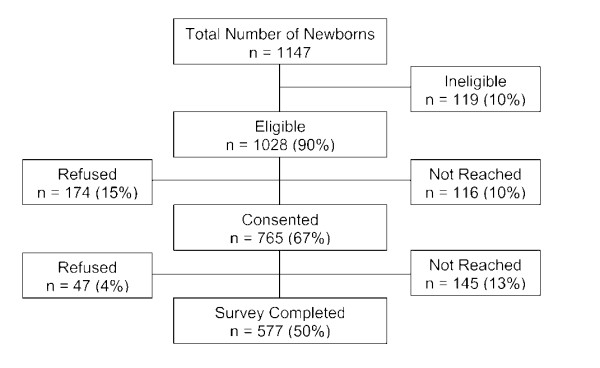
**Participant flow and follow-up**.

Of the 1028 women eligible to participate, 577 completed the survey giving a response rate of 56% (Figure 1). Participant characteristics are given in Table 1. The majority of the mothers were over 31 y. One third of the infants were of European ethnicity and another one third was Chinese. Among those who responded to the annual family income question (*n *= 454), almost half had an income greater than $80,000. Participants were generally well educated with 87% having completed some post-secondary education.

**Table 1:** Participant characteristics

Characteristic	% (*n*)
Maternal Age	

< 31	33.3 (192)

≥31	66.7 (385)

Baby's ethnicity	

European	36.7 (212)

Chinese	31.2 (180)

Other^1^	32.1 (185)

Annual family income	

<$40,000	19.6 (113)

$40,000-59,000	11.3 (65)

$60,000-80,000	10.4 (60)

>$80,000	37.4 (216)

Unknown^2^	21.3 (123)

Education	

< High school	0.3 (2)

Some and completed high school	12.5 (72)

Some trade/vocational training and college/university	8.8 (51)

Completed trade/vocational training and college/university	78.3 (452)

Parity	

Primipara	52.0 (300)

Multipara	48.0 (277)

Baby's gender	

Male	50.6 (292)

Female	49.4 (285)

Marital status	

Single	4.9 (28)

Married	85.6 (494)

Common-law	8.5 (49)

Other	1.0 (6)

^1^South Asian 42.0% (63), South East Asian 42.7% (64), Korean 6.0% (9), Japanese 9.3% (14), Aboriginal 25.7% (9), Black 22.9% (8), Middle Eastern 42.9% (15), Iranian 2.9% (1), Afghan 2.9% (1), Turkish 2.9% (1)

^2^Do not know and do not want to say

The breastfeeding initiation rate was 99% (*n *= 570) and by 2 months of age over 40% of women were exclusively breastfeeding, using the WHO definition (2). Table 2 displays feeding practices and vitamin D supplementation practices at 2 months of age. Nearly 90% of infants were still receiving some breast milk. Over half received only breast milk in the past week; about one third received a mixture of breast milk and formula ranging from ≤2 to > 4 feedings of other liquids/food per day; and about 10% received only infant formula. Of the 577 infants, about 80% were supplemented with vitamin D at the time mothers were surveyed. Rates of vitamin D supplementation were significantly higher in those receiving only breast milk in the past week than those who received both breast milk and formula, which in turn were higher than exclusively formula fed babies. Within the mixed breast milk and formula group, infants receiving < 50% breast milk were less likely to be supplemented with vitamin D than those receiving 50% or more breast milk (*P *< 0.001). The proportion of infants receiving less than 300 IU vitamin D per day (supplement and/or formula) was higher among infants receiving only breast milk than among those fed mixed breast milk and formula. However, within this group there was no difference in the percentage of infants receiving less than 300 IU per day.

**Table 2:** Feeding practices and vitamin D supplementation of infants aged two months

Feeding practice	Feeding Practice	Vitamin D Supplement	**< 300 IU/d Vitamin D**^**5**^
	
	% (*n*)	% yes (*n*)	% (*n*)
Total	100.0 (577)	79.9 (461)	28.9 (167)

All breast milk^1^	57.4 (331)	91.2 (302)^a^*	33.5 (111)^a^

Mixed breast milk and formula	32.2 (186)	79.0 (147)^b^	22.0 (41)^b^

≥75% breast milk^2^	53.8 (100)	86.0 (86)	21.0 (21)

50- < 75% breast milk^3^	20.4 (38)	84.2 (32)	26.3 (10)

< 50% breast milk^4^	25.8 (48)	60.4 (29)	20.8 (10)

Infant formula	10.4 (60)	20.0 (12)^c^	25.9 (15)^ab^

^1^Only breast milk in the past week

^2 ^≤2 feedings of other liquids/food per day

^3^3-4 feedings of other liquids/food per day

^4 ^> 4 feedings of other liquids/food per day

^5^Total daily vitamin D intake from supplement and formula

*Rows showing different superscripts are significantly different from each other; χ^2 ^*P *< 0.001

**Figure 2:**
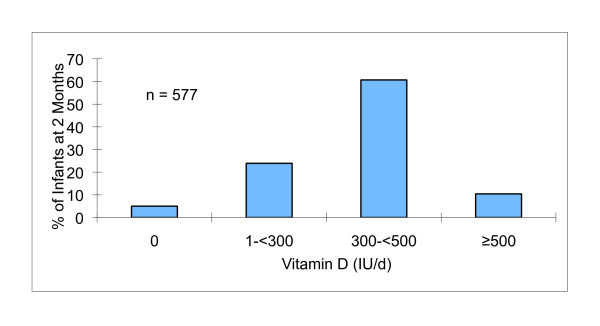
**Daily total vitamin D intake from supplements and infant formula at two months of age**.

About 5% of the infants did not receive any vitamin D from supplements and/or infant formula and 61% had a total vitamin D intake of 300- < 500 IU/d (Figure 2). Approximately 10% received 500 IU or more per day with the highest intake being 1130 IU/d (1000 IU from the supplement). Of those infants who were given vitamin D supplements, 80% received D-Drops^® ^(a concentrated formula that provides 400 IU in a single drop) and 16% received D-Vi-Sol^® ^(a formula that provides 400 IU per 1 mL). As shown in Table 3, over 90% of participants recalled receiving one or more recommendations to give their infant a vitamin D supplement. Public health nurses and physicians were the most frequent sources of this recommendation. Women who did use a supplement were asked to respond to an open question on why they chose to do so. The most common reasons were related to the inadequate amounts of vitamin D in breast milk, that a supplement had been recommended, that vitamin D had health benefits for the infant, and that lack of sunlight exposure meant supplementation was needed. Women who did not provide a supplement were asked to choose from a list on why they did not. Among those providing a response, the most common reasons were that the infant was being given formula, or that they did not think supplementation was necessary.

**Table 3:** Advice and decisions on vitamin D supplementation

	% (n)
Did anyone ever recommend a vitamin D supplement?	

Yes	92.2 (532)

No	7.8 (45)

Who recommended the supplement? (*n *= 532)^a^	

Public health nurse	80.2 (426)

Doctor	69.7 (370)

Midwife	9.6 (51)

Dietitian/pharmacist	5.1 (27)

Family member or friend	19.4 (103)

Other^1^	7.7 (46)

Reasons for providing a vitamin D supplement (*n *= 452)^a^	

Not in breast milk/I am breastfeeding	43.6 (197)

It was recommended	30.3 (137)

Health benefits for infant	28.3 (128)

Lack of sunlight/northern climate	21.0 (95)

Other^2^	13.1 (59)

Reasons for not supplementing vitamin D (*n *= 116)^a^	

Baby is being fed formula	35.3 (41)

I didn't know to give	10.3 (12)

I don't think the baby needs it	19.0 (22)

Forgot to give	3.4 (4)

Baby did not tolerate (vomit/spit up)	0.9 (1)

Other^3^	5.2 (6)

No response	28.4 (33)

^a^Multiple responses possible

^1^Reading, television, internet, South Community Birth Program, Baby's Best Chance, prenatal class, lactation clinic, natural path, doula, specialist, from first child

^2^Knew from first baby, read about it, did not know why, it is important

^3^Confused with the recommended dose, will purchase later, will ask doctor, first baby did not tolerate, had not started yet

In multiple regression analysis of infants receiving only breast milk in the week prior to the survey (*n *= 331), only parity was associated with vitamin D supplementation. Primiparas were more likely to supplement their infants than multiparas (95% versus 84%; *P *= 0.03). Table 4 shows the results of the logistic regression of selected variables associated with vitamin D supplementation in infants who received greater than 50% of their feeds from breast milk (*n *= 517). None of the variables studied was significantly associated with supplementation, although there was a tendency for a higher rate of vitamin D supplementation in infants from families in the two higher income categories than those in the lowest income category.

**Table 4:** Percentages and multivariate adjusted odds ratio (and 95% CIs) for infant vitamin D supplementation in infants receiving greater than 50% of feeds from breast milk by select characteristics (*n *= 420)

Characteristic	% (*n*)	OR (95% CI)	*p*-value
Maternal Age			

< 31 y	87.6 (134)	1.00	

≥31 y	89.9 (286)	1.28 (0.65, 2.50)	0.479

Baby's ethnicity			

European	88.9 (160)	1.00	

Chinese	89.6 (129)	1.34 (0.62, 2.86)	0.456

Other	89.1 (131)	1.32 (0.62, 2.80)	0.469

Annual family income			

<$40,000	83.1 (74)	1.00	

$40,000-80,000	92.2 (95)	2.51 (0.98, 6.41)	0.054

>$80,000	91.4 (170)	2.24 (0.94, 5.30)	0.068

Non-responder^1^	87.1 (81)	1.35 (0.58, 3.14)	0.488

Education			

Less than completed trade/vocational training and college/university	87.0 (87)	1.00	

Completed trade/vocational training and college/university	89.8 (333)	0.99 (0.48, 2.04)	0.984

Parity			

Primipara	90.5 (219)	1.00	

Multipara	87.8 (201)	0.69 (0.37, 1.29)	0.244

Note: *CI *confidence interval, *OR *multivariate adjusted odds ratio

^1^Do not know and do not want to say

## Discussion

In this study we build on previous Canadian observations indicating high rates of breastfeeding and vitamin D supplementation, and provide new information regarding the dose of vitamin D provided, and mothers' reasons for choosing to supplement or not supplement. Almost 60% of infants had received only breast milk in the week prior to the survey and of these greater than 90% had received vitamin D supplements. This is much higher than the 67% vitamin D supplementation rate reported in a 2007-2008 survey for Canadian women who had exclusively breastfed an infant in the past 5 years [17]. In that study, the supplementation rate in BC was marginally higher than the national average (70%), but still well below the rate in our study. Our rates of vitamin D supplementation are comparable to those reported more recently in one Montreal hospital where 98% of exclusively breastfed (WHO definition) infants had been supplemented with vitamin D at some point during the first 6 months [18]. Data from the Infant Feeding Practices Study II (2005-2007) suggest that US vitamin D supplementation rates are markedly lower than in Canada; 43% of infants were breastfed at 2 months and of these only 10% were receiving vitamin D [21]. However, the American Academy of Pediatrics only began recommending infant supplementation in November 2008, whereas the Canadian recommendation has been present in some form since 1967. Further, breastfeeding rates have been historically higher in Canada than in the US and Canada's higher latitude may have created a greater impetus for infant supplementation in this country. As expected infant supplementation at 2 months was lower amongst those receiving mixed feeds (79%) and lower still in infants receiving only infant formula (20%). In Montreal, an apparently higher 88% of mixed feeders had received supplemental vitamin D; however, this was anytime during the first 6 months [18]. In the US study, amongst mixed feeders, only 5% were receiving vitamin D supplements at 2 months of age [21]. Despite a high proportion of breastfed infants receiving vitamin D supplements, one third were not receiving at least 300 IU/d; mainly because of less than daily supplement administration. Although we report less frequent vitamin D supplementation rates among infants who were receiving mixed breast milk and formula in the week before the survey than among those fed only breast milk (79% versus 91%), fewer of the infants receiving mixed feeding had vitamin D intakes below 300 IU/d vitamin D (22%, versus 33.5% in the fully breastfed group). Further, within the group receiving mixed feeds, as the amount of formula increased infant supplementation dropped; however, the percentage of those receiving less than 300 IU/d remained constant at around 20%. This was not unexpected as formula is fortified with vitamin D and it would take about 700 ml of formula to achieve an intake of 300 IU/d. Consuming less than 700 ml/d also explains why 25% of formula fed infants failed to achieve this intake. Using a stricter cut-point of 400 IU/d, the Montreal researchers reported that 74% of exclusively breastfed infants and 51% receiving mixed feeds achieved an intake of 400 IU/d at 6 months [18].

Up to a third of infants not achieving 300 IU/d vitamin D and even less achieving the recommendation of 400 IU/d may appear high. However, only 5% of infants were receiving no vitamin D. Further, it is acknowledged that the evidence base used to derive the infant recommendation is limited. Serum 25-hydroxyvitamin D (25OHD) concentration is the best indicator of vitamin D status. Although controversial the US Institute of Medicine recently affirmed a 25OHD of 50 nmol/L as desirable in all age groups including infants [19]. Greer et al. [22] showed that breastfed infants (*n *= 9) receiving 400 IU had mean 25-hydroxyvitamin D concentrations of 95 nmol/L after 12 weeks. More recently, infants randomized to 250 or 500 IU per day (*n *= 20 per group) at birth achieved mean (95% CI) 25OHD concentrations of 139 (114-164) and 151 (126-176) nmol/L, respectively after 6 weeks [20]. Thus, it appears that the recommended intake of 400 IU exceeds the requirements of almost all infants, perhaps by a considerable margin. There have been reports of infant overdosing with vitamin D in the US resulting in the Food and Drug Administration issuing a warning of the potential risk of overdosing infants with liquid vitamin D [23]. In our study only one infant was receiving greater than the upper limit for vitamin D of 1000 IU suggesting this was not a problem.

Among caregivers there was generally good awareness of the need to supplement and why it was important. For example, caregivers indicated that they used a supplement because vitamin D was not present in adequate amounts in breast milk and/or that sunlight exposure was limited or not recommended; and many women who used formula appeared to be aware that supplementation wasn't required. Over 90% of caregivers recalled receiving advice primarily from public health nurses and doctors to supplement with vitamin D, which may explain the high rates of supplementation. In a Seattle study [24], parents who reported that their child's pediatrician recommended vitamin D were 8 times more likely to provide the supplementation than parents whose child's pediatrician did not. However, only a third of parents recalled receiving any recommendation and of these under half supplemented with vitamin D. In contrast to our study, where < 5% of caregivers thought supplementation was unnecessary, 67% of parents in the Seattle study believed that supplementation was unnecessary because breast milk has all needed nutrition.

Multivariate regression revealed little in the way of predictors of supplement use. There was a non-significant tendency for family incomes less than $40,000 to be associated with lower rates of supplementation. However, vitamin D supplements cost less than $40 for 6 months and cost of the supplements was not given as a reason for not supplementing. Interestingly 80% of caregivers reported giving their infants D-Drops^® ^versus only 16% who supplemented with D-Vi-Sol^® ^. The reason for the popularity of D-Drops^® ^may be their ease of administration requiring only a single drop that can be placed on the mother's breast prior to nursing, versus the need to use a dropper to administer D-Vi-Sol^® ^[25].

A strength of our study was that we had access to a database that contained the names of nearly all infants born in Vancouver and Richmond over the study period. Also, we sampled an ethnically diverse population where breastfeeding rates are high relative to the rest of North America. Finally, data were collected prospectively at 2 months rather than relying on recall of up to 5 years in one study. In studies of this type, selection bias is always an important consideration. For example, people who choose to participate versus those who do not, may be more educated and of higher socioeconomic status and thus more likely to breastfeed and supplement with vitamin D. Thus a limitation of our study is that we had only a moderate response rate of 56%. Unfortunately, we do not have any data on our non-responders and there are no representative data on pregnant women in Vancouver and Richmond to compare our results with. However, with respect to ethnicity [26], education [27] and family income [28] our sample compares well with 2006 census data for women from Vancouver and Richmond. Further, only half of the non-responders refused participation, while the other half could not be contacted initially or at follow-up. Because the survey was conducted in the summer months it appeared that many women were away on vacations or staying with family outside the area. We acknowledge that our findings cannot be extrapolated to the rest of Canada or even BC. Breastfeeding rates are higher in BC than elsewhere and Vancouver and Richmond have a unique ethnic mix not present in the rest of the province or Canada. Second, we only sampled at 2 months; this was an intentional decision but more data are needed on older infants. In the US, supplementation rates remained relatively constant out to 12 months; however, in this study both breastfeeding rates and infant supplementation were much lower than ours [21]. In Montreal, of all supplemented breastfed infants around a third had stopped taking the supplement by 6 months [18]. More data are needed on older infants especially around the time of introduction of solids and as breastfeeding rates drop with age.

## Conclusions

Breastfeeding rate was high among this group of mothers. About 90% of the infants received breast milk and 57% were exclusively breastfed at 2 months. Over 60% of the infants had a total vitamin D intake of 300- < 500 IU/d from supplements and formula. The vitamin D supplementation rate was 80%. There was good awareness of the need to supplement with vitamin D and the reasons why. Most parents were advised to give their infants vitamin D supplement by public health nurses and doctors. Overall it appears that the level of knowledge translation about the importance of vitamin D is high. This points to the success of Vancouver Coastal Heath's system of universal contact by public health nurses, especially for providing breast feeding support and also for providing key public health messages such as the use of vitamin D. Future studies are needed to monitor vitamin D supplement rates of older infants and toddlers.

## Competing interests

The authors declare that they have no competing interests.

## Authors' contributions

BC and TG were responsible for the conception of the study and obtaining funds. All authors had a role in the design, acquisition of data, analysis and interpretation of data. BC, TG, SB, and WL drafted the initial manuscript. All authors read and approved the final manuscript.

## Pre-publication history

The pre-publication history for this paper can be accessed here:

http://www.biomedcentral.com/1471-2458/11/905/prepub
